# Impaired sensitivity to thyroid hormones and carotid plaque in patients with coronary heart disease: A RCSCD-TCM study in China

**DOI:** 10.3389/fendo.2022.940633

**Published:** 2022-09-27

**Authors:** Yijia Liu, Zhu Li, Tong Yang, Lin Li, Lu Yu, Fanfan Liu, Tongyao Ni, Shan Gao, Chunjie Li, Rongrong Yang, Chunquan Yu

**Affiliations:** ^1^Department of Graduate School, Tianjin University of Traditional Chinese Medicine, Tianjin, China; ^2^Department of Emergency, Tianjin Chest Hospital, Tianjin, China

**Keywords:** coronary heart disease, carotid plaque, thyroid hormone sensitivity, association, TFQI, PTFQI, resistance to thyroid hormone

## Abstract

**Context:**

Previous studies on the association between thyroid function and carotid plaque have shown contradictory results, which may be attributable to the sensitivity to thyroid hormone indices. This study aimed to analyze the association between thyroid hormone sensitivity and risk of carotid plaque in patients with coronary heart disease (CHD) and further explore this association according to sex, age, smoking, and drinking status.

**Methods:**

This large-scale, multi-center, retrospective, cross-sectional study included 6679 patients with CHD (age 35–75). Central sensitivity to thyroid hormone was evaluated by the thyroid feedback quantile-based index (TFQI), parametric thyroid feedback quantile-based index (PTFQI), thyroid-stimulating hormone index (TSHI), and thyrotroph thyroxine resistance index (TT4RI). Peripheral sensitivity to thyroid hormone was assessed by free triiodothyronine/free thyroxine (FT3/FT4) ratio. Taking no carotid plaque as a reference, this study used logistic regression to analyze the association between central and peripheral thyroid hormone sensitivity and carotid plaque in patients with CHD.

**Results:**

Of the 6679 patients with CHD, 4843 (72.50%) had carotid plaque. In the multi-adjusted models, the TFQI (odds ratio [OR]: 1.50; 95% confidence interval [CI]: 1.26–1.78; *P* < 0.001), PTFQI (OR: 1.76; 95% CI: 1.46–2.12; *P* < 0.001), TSHI (OR: 1.21; 95% CI: 1.10–1.33; *P* < 0.001), and TT4RI (OR: 1.00; 95% CI: 1.00–1.01; *P* = 0.003) were positively associated with the risk of carotid plaque. Compared with that in females and people > 60 years, the OR value for carotid plaque was higher in males and people ≤ 60 years. Similarly, smokers and drinkers had higher OR values for carotid plaque than non-smokers and non-drinkers. Conversely, FT3/FT4 ratio (OR: 0.75; 95% CI: 0.70–0.81; *P* < 0.001) was negatively associated with carotid plaque, and the OR value for carotid plaque was lower in males, patients ≤ 60 years, smokers, and drinkers.

**Conclusion:**

This study showed that thyroid hormone sensitivity is significantly associated with carotid plaque in patients with CHD. This association is more significant in males, patients ≤ 60 years, smokers, and drinkers.

## Graphical Abstract

Images are from Biorender.com.
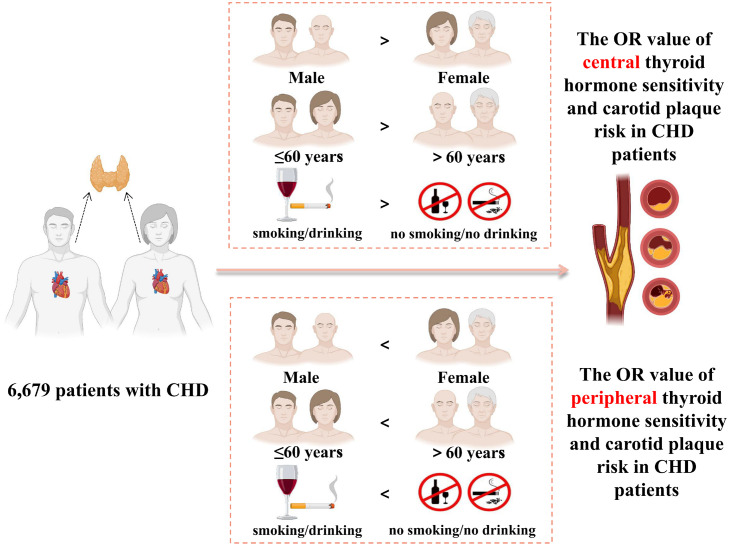

## Introduction

Cardiovascular disease is one of the most common causes of death worldwide (1), seriously affecting the patient’s quality of life and longevity (2). The risk of cardiovascular and cerebrovascular events is generally increased in patients with coronary heart disease (CHD), and most cardiac deaths are caused by CHD secondary to coronary atherosclerosis (3, 4). Carotid plaque burden has been proven to be a good marker for cardiovascular or cerebrovascular disease events (5, 6), and it is also clinically relevant in the CHD population compared to in the healthy population.

CHD is closely related to thyroid hormones, and patients with CHD are often found to have abnormal levels of thyroid hormones (7, 8). Abnormal thyroid hormone levels are associated with increased systemic vascular resistance, decreased cardiac contractility, reduced cardiac output, and accelerated atherosclerosis and CHD owing to hypercholesterolemia and diastolic hypertension (9). A prospective cohort study reported that the greater the changes in thyroid hormone levels in euthyroid, middle-aged, or older participants, the higher the risk of carotid atherosclerosis (10). Sakamaki et al. (11) believed that thyroid stimulating hormone (TSH) was independently associated with carotid plaque, especially when the TSH level was ≥2.5 μIU/mL. However, another cross-sectional study (12) found that TSH and free thyroxine (FT4) were not significantly associated with carotid plaque. These conflicting results seem to be common. In addition, almost all previous analyses focused solely on TSH, free triiodothyronine (FT3), and FT4 levels to assess the risk of carotid plaque. At the same time, indices of thyroid hormone sensitivity can also be used to evaluate the complex interactions between FT3, FT4, and TSH, which can provide a new reference marker for thyroid function. However, no studies have investigated the association between thyroid hormone sensitivity and carotid plaque in patients with CHD. Although previous studies have investigated the prevalence of carotid plaque according to sex, age, and smoking and drinking status (13, 14), the results were inconsistent. Owing to these inconsistent results, the association between carotid plaque and sex, age, and smoking and drinking status remains controversial.

Therefore, this study aimed to investigate the association between central and peripheral thyroid hormone sensitivity and carotid plaque in patients with CHD, to further explore the association with sex, age, and smoking and drinking status, and to provide a basis for management of patients with clinical CHD.

## Materials and methods

### Study population

**Figure 1:**
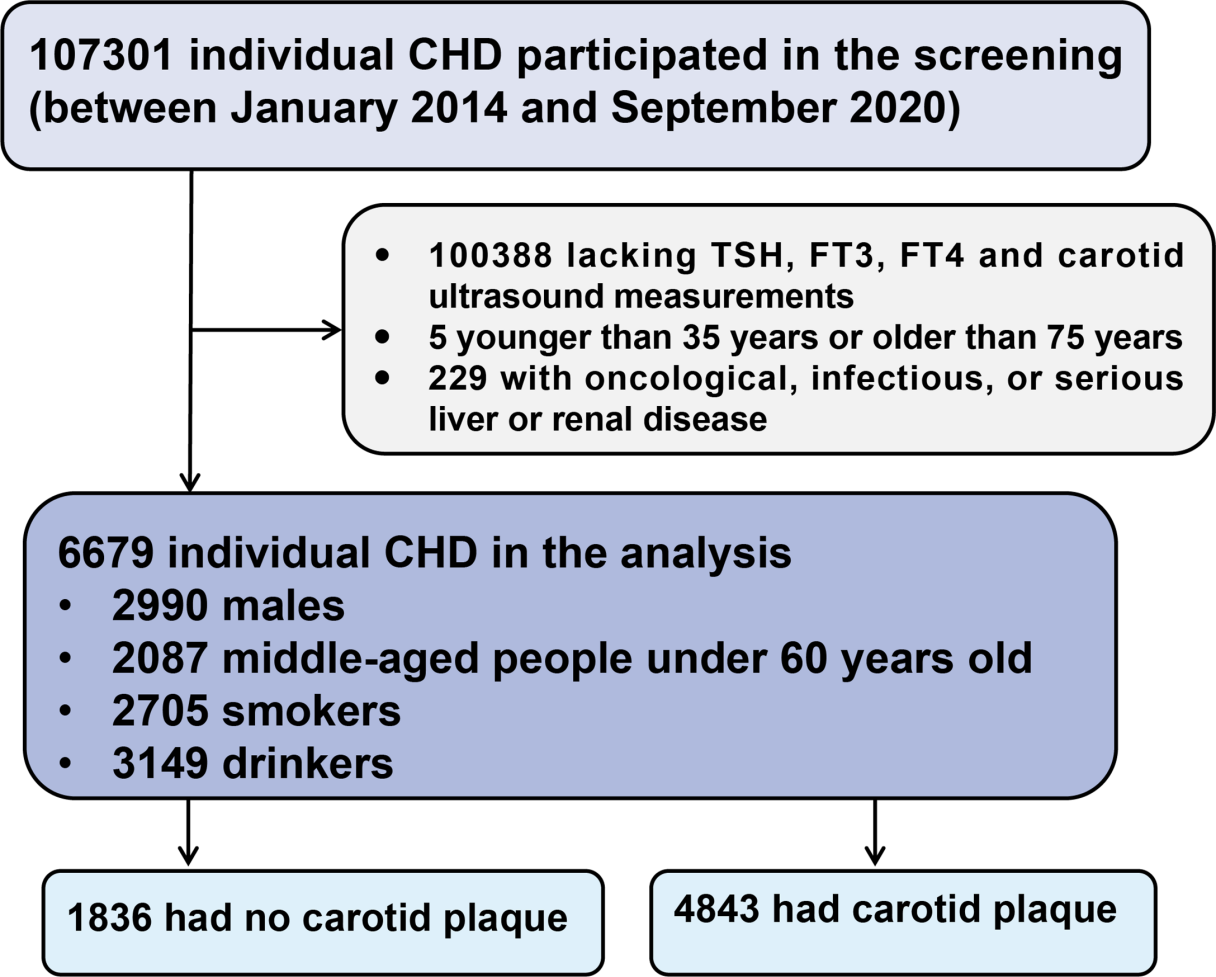
Flow chart of the study population. CHD, coronary heart disease; TSH, thyroid stimulating hormone; FT3, free triiodothyronine; FT4, free thyroxine.

This large-scale, multi-center, retrospective, cross-sectional study included 107301 CHD patients hospitalized in six Tianjin hospitals between January 1, 2014, and September 30, 2020 (15). Inclusion criteria were: i) patients with CHD meeting the diagnosis of the International Classification of Diseases 10th revision (ICD-10) codes (I20, I24-I25, and I49-I50), and ii) hospital admission during the defined period. Patients were excluded from the analyses if they: i) lacked TSH, FT3, FT4, and carotid ultrasound measurements, ii) were younger than 35 years or older than 75 years, or iii) had oncological, infectious, or severe liver or renal disease. Therefore, a total of 6679 individuals were included in the current analyses. A flowchart of the patient recruitment process is shown in **Figure 1**.

This study was approved by the Ethics Committee of Tianjin University of Traditional Chinese Medicine (TJUTCM-EC20190008) and was registered in the Chinese Clinical Trial Registry (ChiCTR-1900024535) and Clinical Trials.gov (NCT04026724).

### Data collection

Trained medical personnel collected data of age, sex, and smoking and drinking status by means of a standard structured questionnaire (16). The question regarding smoking/drinking habits had two response options: 1) “no;” 2) “yes.” “Never smoked/non-smoker” and “no/mild drinking” were defined as “no,” “current smoker/former smoker” and “heavy drinking” were defined as “yes” (17, 18). The systolic and diastolic blood pressures (SBP and DBP) were measured by trained physicians using an electronic device. Hypertension was defined as SBP  ≥ 130  mmHg or DBP  ≥ 80 mmHg (19). Rheumatological diseases included rheumatoid arthritis (ICD-10 codes M05-06), psoriatic arthritis (ICD-10 codes L40), ankylosing spondylitis (ICD-10 codes M45), vasculitis (ICD-10 codes I77), systemic lupus erythematosus (ICD-10 codes M32), sjögren (ICD-10 codes M35), poly/dermatomyositis (ICD-10 codes M33), systemic sclerosis (ICD-10 codes M34) (20, 21). Thyroid diseases (ICD-10 codes E00-E07) included hyperthyroidism, hypothyroidism, thyroiditis, thyroid cyst, thyrophyma, or thyroid nodule (22). Autoimmune thyroid diseases (AITD) included Graves’ disease and Hashimoto thyroiditis (23, 24).

Fasting venous blood samples were obtained from all participants on the second day of hospitalization. Glycated hemoglobin (HbA1c), triglyceride (TG), total cholesterol (TC), high-density lipoprotein cholesterol (HDL-C), low-density lipoprotein cholesterol (LDL-C), and C-reactionprotein (CRP) levels were measured using an automatic hematology analyzer. Standard laboratory procedure for quality control were strictly followed (16). Type 2 diabetes was defined as an elevated HbA1c  level ≥ 6.5% (25). Dyslipidemia was defined as TG level of ≥ 2.3 mmol/L, TC level of ≥ 6.2 mmol/L, HDL level of ≤ 1.0 mmol/L, or LDL level of ≥ 4.1 mmol/L (26).

The concentrations of TSH, FT3, and FT4 were measured with the automated immunochemiluminescent assay kits. The reference ranges of TSH, FT4, and FT3 were 0.35–4.94 mIU/L, 9.01–19.05 pmol/L, and 2.63–5.70 pmol/L, respectively. Indices reflecting central thyroid hormone sensitivity, including thyroid feedback quantile-based index (TFQI), parametric thyroid feedback quantile-based index (PTFQI), thyroid-stimulating hormone index (TSHI), and thyrotroph thyroxine resistance index (TT4RI), were calculated according to previous studies as follows:


TSHI=ln TSH(mIU/L)+0.1345*FT4(pmol/L)

(27)


TT4RI=FT4(pmol/L)*TSH(mIU/L)

(28)


TFQI=cdf fT4−(1−cdf TSH),

PTFQI=φ((fT4−μfT4)/σfT4)−(1−φ((ln TSH−μln TSH)/σln TSH))

(29)

For TSHI, TT4RI, TFQI and PTFQI, the higher the values, the lower the central sensitivity to thyroid hormones. FT3/FT4 ratio was calculated to evaluate peripheral thyroid hormone sensitivity. Higher FT3/FT4 indicates higher peripheral thyroid hormone sensitivity.

Using an ultrasound diagnostic system, trained and certified physicians performed carotid artery ultrasounds. Images were obtained at the common carotid artery, internal carotid artery, and carotid bifurcation in the supine position. The carotid arteries were carefully scanned in multiple directions using b-mode imaging. The carotid intima-media thickness (CIMT) was defined as the average IMT value of the right and left common carotid arteries (30). Carotid artery color-Doppler was analyzed by professional physicians according to Doppler ultrasound results, and the number and echo properties of carotid plaques were recorded. The number of carotid plaques was classified as single (*n* = 1) or multiple (*n* ≥ 2). The echo properties of carotid plaques included hypoechoic, isoechoic, hyperechoic, and mixed. Strict quality control procedures were implemented for image acquisition and analysis, and inter-laboratory quality evaluations were performed by certified personnel.

### Statistical analyses

The Chi-squared (χ^2^) and Mann–Whitney U test were used to compare the characteristics of the participants in the different groups. Using logistic regression, the odds ratios (ORs) and 95% confidence intervals (CIs) of carotid plaque were estimated for the thyroid hormone indices. Age, sex, TC, TG, HDL-C, LDL-C, SBP, DBP, HbA1c, smoking, and drinking were potential confounders. Additionally, we performed the following sensitivity analysis: restricting analyses to participants with non-thyroid disease; excluding participants with AITD or rheumatological disease. Stratification was performed by sex, age categories (cutoff age: 60 years), and smoking and drinking status. Missing values for SBP (*n* = 17), DBP (*n* = 18), hypertension (*n* = 18), TC (*n* = 265), TG (*n* = 265), HDL-C (*n* = 264), LDL-C (*n* = 264), dyslipidemia (*n* = 264), HbA1c (*n* = 530), type 2 diabetes (*n* = 530), smoking status (*n* = 24), drinking status (*n* = 50), CIMT (*n* = 2114), carotid plaque echogenicity (*n* = 80), and CRP (*n* = 3161) were imputed using multiple imputation methods. All statistical analyses were performed using SPSS 24.0 (IBM Corp, New York, NY, USA).

## Results

### Baseline characteristics

The baseline clinical data of participants are presented in **Table 1**. A total of 6679 participants were included for data analysis, including 2990 males (44.80%) with an average age of 64.00 ± 8.00 years. Among them, 4843 patients had carotid plaque (72.51%). Compared with patients without carotid plaque, patients with carotid plaque were more likely to be older females, smokers, and drinkers and were more likely to develop hypertension, diabetes, and hyperlipidemia. In addition, participants with carotid plaque tended to have higher levels of TFQI, PTFQI, TSHI, and TT4RI, while FT3/FT4 was lower.

**Table 1:** General characteristics of study participants.

Characteristic	Total (*n*=6679)	No carotid plaque (*n*=1836)	Carotid plaque (*n*=4843)	*P*-value
Sex, *n* (%)				<0.001
Male	2990 (44.77)	580 (31.59)	2410 (49.76)	
Female	3689 (55.23)	1256 (68.41)	2433 (50.24)	
Age, years, median (IQR)				<0.001
Total	64 (59,70)	60 (54,66)	66 (61,71)	
≤60	55 (52,58)	54 (49,57)	56 (53,59)	
>60	67 (64,71)	66 (63,70)	68 (64,72)	
SBP, mmHg, median (IQR)	140 (127,156)	136 (122,150)	141 (130,158)	<0.001
DBP, mmHg, median (IQR)	83 (76,90)	83 (77,91)	82 (76,90)	0.058
Hypertension, n (%)	5561 (83.26)	1456 (79.30)	4105 (84.76)	<0.001
HbA1c, %, median (IQR)	6.00 (5.60,6.90)	5.80 (5.50,6.40)	6.10 (5.60,7.10)	<0.001
Type 2 diabetes, n (%)	2330 (34.89)	443 (24.13)	1887 (38.96)	<0.001
TG, mmol/L, median (IQR)	1.45 (1.04,2.08)	1.41 (1.00,2.05)	1.46 (1.05,2.09)	0.016
TC, mmol/L, median (IQR)	4.60 (3.87,5.38)	4.61 (3.99,5.33)	4.60 (3.83,5.39)	0.202
HDL,C, mmol/L, median (IQR)	1.08 (0.92,1.28)	1.13 (0.96,1.32)	1.06 (0.90,1.26)	<0.001
LDL,C, mmol/L, median (IQR)	2.76 (2.14,3.41)	2.73 (2.15,3.28)	2.78 (2.14,3.45)	0.016
Dyslipidemia, n (%)	3575 (53.53)	848 (46.19)	2727 (56.31)	<0.001
Smoking, n (%)	2705 (40.50)	484 (26.36)	2221 (45.86)	<0.001
Drinking, n (%)	3149 (47.15)	643 (35.02)	2506 (51.74)	<0.001
TSH, mIU/L, median (IQR)	1.86 (1.23,4.40)	1.91 (1.29,2.85)	1.84 (1.22,2.86)	0.101
FT3, pmol/L, median (IQR)	2.74 (2.40,3.71)	2.63 (2.34,3.05)	2.83 (2.42,3.83)	<0.001
FT4, pmol/L, median (IQR)	1.11 (0.96,14.70)	1.03 (0.93,1.26)	1.19 (0.97,15.21)	<0.001
TFQI, median (IQR)	-0.01 (-0.30,0.29)	-0.08 (-0.36,0.15)	0.02 (-0.27,0.33)	<0.001
PTFQI, median (IQR)	-0.59 (-0.34,0.30)	-0.17 (-0.38,0.08)	-0.01 (-0.32,0.36)	<0.001
TSHI, median (IQR)	1.07 (0.53,1.44)	0.89 (0.44,1.35)	1.14 (0.57,1.47)	<0.001
TT4RI, median (IQR)	3.09 (1.53,24.25)	2.27 (1.40,5.29)	4.13 (1.62,27.81)	<0.001
FT3/FT4, median (IQR)	2.16 (0.27,2.63)	2.40 (1.82,2.74)	1.96 (0.26,2.58)	<0.001

Data are presented as median (interquartile) or number (proportion, %). *P*-values were calculated using Mann-Whitney U test. IQR, interquartile range; SBP, systolic blood pressure; DBP, diastolic blood pressure; HbA1c, glycated hemoglobin; TC, total cholesterol; TG, triglycerides; HDL-C, high-density lipoprotein cholesterol; LDL-C, low-density lipoprotein cholesterol; TSH, thyroid-stimulating hormone; FT3, free triiodothyronine; FT4, free thyroxine; TFQI, thyroid feedback quantile-based index; PTFQI, parametric thyroid feedback quantile-based index; TSHI, TSH index; TT4RI, thyrotroph thyroxine resistance index.

### Association between thyroid hormone sensitivity and carotid plaque

Three logistic regression models were constructed to assess the effect of thyroid hormone sensitivity on carotid plaque (**Table 2**). In the multi-adjusted models, it was found that TFQI (OR: 1.50; 95% CI: 1.26–1.78; *P* < 0.001), PTFQI (OR: 1.76; 95% CI: 1.46–2.12; *P* < 0.001), TSHI (OR: 1.21; 95% CI: 1.10–1.33; *P* < 0.001), and TT4RI (OR: 1.00; 95% CI: 1.00–1.01; *P =* 0.003) were positively associated with the risk of carotid plaque, but FT3/FT4 (OR: 0.75; 95% CI: 0.70–0.81; *P* < 0.001) was negatively associated with carotid plaque, which was consistent with unadjusted results. ORs for the fourth versus the first quartile of TFQI, PTFQI, TSHI, TT4RI, and FT3/FT4 were 2.08 (95% CI: 1.75–2.47) (*P*_trend_ < 0.001), 2.36 (95% CI: 1.98–2.81) (*P*_trend_ < 0.001), 1.83 (95% CI: 1.55–2.17) (*P*_trend_ < 0.001), 2.31 (95% CI: 1.94–2.75) (*P*_trend_ < 0.001), and 0.38 (95% CI: 0.32–0.46) (*P*_trend_ < 0.001), respectively, for carotid plaque. This study excluded participants with thyroid disease, and further evaluated the association between thyroid hormone sensitivity and carotid plaque in participants with non-thyroid disease, and the results showed no significant change (**Table 2**). And there was no significant change in the association between thyroid hormone sensitivity and carotid plaque when participants with AITD, or rheumatological disease were excluded (**Supplemental Tables S1**, **S2**). The associations of thyroid hormone sensitivity with the number and echo properties of carotid plaques and CIMT were further evaluated. The results show that the association remained significant (**Supplemental Tables S3**–**S5**). In addition, when the present study supplemented CRP as a potential confounder for analysis, the association between thyroid hormone sensitivity and carotid plaque did not alter much in the fully adjusted model (**Supplemental Table S6**).

**Table 2:** Association between thyroid hormone sensitivity and carotid plaque.

Population	Variables	Carotid plaque					
		OR (95% CI)^1^	*P*-value	OR (95% CI)^2^	*P*-value	OR (95% CI)^3^	*P*-value
CHD (*n*=6679)	TFQI	2.07 (1.78-2.40)	<0.001	1.70 (1.45-1.99)	<0.001	1.50 (1.26-1.78)	<0.001
	Q1	Reference		Reference		Reference	
	Q2	1.11 (0.95-1.30)	0.19	1.03 (0.87-1.21)	0.77	1.03 (0.88-1.22)	0.7
	Q3	1.51 (1.28-1.78)	<0.001	1.36 (1.15-1.61)	<0.001	1.31 (1.10-1.55)	0.002
	Q4	2.08 (1.75-2.47)	<0.001	1.68 (1.41-2.00)	<0.001	1.45 (1.20-1.76)	<0.001
	*P*_trend_		<0.001		<0.001		<0.001
	PTFQI	2.44 (2.09-2.85)	<0.001	1.97 (1.68-2.32)	<0.001	1.76 (1.46-2.12)	<0.001
	Q1	Reference		Reference		Reference	
	Q2	0.88 (0.75-1.03)	0.12	0.86 (0.74-1.02)	0.08	0.89 (0.76-1.05)	0.17
	Q3	1.57 (1.33-1.85)	<0.001	1.39 (1.17-1.65)	<0.001	1.37 (1.15-1.63)	<0.001
	Q4	2.36 (1.98-2.81)	<0.001	1.91 (1.60-2.30)	<0.001	1.74 (1.41-2.14)	<0.001
	*P*_trend_		<0.001		<0.001		<0.001
	TSHI	1.43 (1.31-1.55)	<0.001	1.30 (1.19-1.41)	<0.001	1.21 (1.10-1.33)	<0.001
	Q1	Reference		Reference		Reference	
	Q2	0.99 (0.84-1.16)	0.87	0.94 (0.80-1.11)	0.48	0.96 (0.81-1.13)	0.58
	Q3	1.72 (1.46-2.04)	<0.001	1.49 (1.25-1.77)	<0.001	1.37 (1.15-1.64)	<0.001
	Q4	1.83 (1.55-2.17)	<0.001	1.53 (1.29-1.82)	<0.001	1.92 (1.61-2.29)	<0.001
	*P*_trend_		<0.001		<0.001		<0.001
	TT4RI	1.01 (1.00-1.02)	<0.001	1.01 (1.01-1.01)	<0.001	1.00 (1.00-1.01)	0.003
	Q1	Reference		Reference		Reference	
	Q2	0.85 (0.72-0.99)	0.04	0.82 (0.70-0.97)	0.02	0.86 (0.73-1.01)	0.07
	Q3	1.63 (1.38-1.92)	<0.001	1.44 (1.22-1.71)	<0.001	1.43 (1.20-1.70)	<0.001
	Q4	2.31 (1.94-2.75)	<0.001	1.87 (1.56-2.24)	<0.001	1.71 (1.39-2.11)	<0.001
	*P*_trend_		<0.001		<0.001		<0.001
	FT3/FT4	0.68 (0.65-0.72)	<0.001	0.74 (0.70-0.78)	<0.001	0.75 (0.70-0.81)	<0.001
	Q1	Reference		Reference		Reference	
	Q2	0.69 (0.57-0.82)	<0.001	0.77 (0.64-0.93)	0.01	0.81 (0.66-0.99)	0.04
	Q3	0.39 (0.33-0.46)	<0.001	0.47 (0.39-0.56)	<0.001	0.53 (0.43-0.65)	<0.001
	Q4	0.38 (0.32-0.46)	<0.001	0.48 (0.40-0.58)	<0.001	0.53 (0.43-0.66)	<0.001
	*P*_trend_		<0.001		<0.001		<0.001
CHD with non-thyroid disease (*n*=4748)	TFQI	2.27 (1.89-2.72)	<0.001	1.87 (1.55-2.25)	<0.001	1.72 (1.40-2.11)	<0.001
	Q1	Reference		Reference		Reference	
	Q2	1.13 (0.95-1.36)	0.180	1.06 (0.88-1.27)	0.572	1.08 (0.90-1.31)	0.416
	Q3	1.64 (1.35-1.98)	<0.001	1.47 (1.21-1.79)	<0.001	1.44 (1.18-1.76)	<0.001
	Q4	2.27 (1.85-2.79)	<0.001	1.84 (1.48-2.28)	<0.001	1.65 (1.31-2.08)	<0.001
	*P*_trend_		<0.001		<0.001		<0.001
	PTFQI	2.72 (2.25-3.28)	<0.001	2.21 (1.82-2.69)	<0.001	2.09 (1.67-2.63)	<0.001
	Q1	Reference		Reference		Reference	
	Q2	0.95 (0.80-1.13)	0.569	0.93 (0.76-1.11)	0.424	0.97 (0.81-1.17)	0.755
	Q3	1.64 (1.35-1.99)	<0.001	1.46 (1.19-1.78)	<0.001	1.48 (1.20-1.81)	<0.001
	Q4	2.72 (2.19-3.38)	<0.001	2.22 (1.78-2.78)	<0.001	2.13 (1.65-2.76)	<0.001
	*P*_trend_		<0.001		<0.001		<0.001
	TSHI	1.56 (1.40-1.74)	<0.001	1.41 (1.26-1.58)	<0.001	1.36 (1.21-1.53)	<0.001
	Q1	Reference		Reference		Reference	
	Q2	1.04 (0.87-1.25)	0.638	1.00 (0.83-1.20)	0.995	1.03 (0.86-1.24)	0.745
	Q3	1.71 (1.41-2.08)	0.001	1.50 (1.23-1.83)	<0.001	1.43 (1.17-1.76)	0.001
	Q4	2.13 (1.73-2.61)	<0.001	1.77 (1.43-2.19)	<0.001	1.67 (1.33-2.09)	<0.001
	*P*_trend_		<0.001		<0.001		<0.001
	TT4RI	1.02 (1.02-1.03)	<0.001	1.02 (1.01-1.02)	<0.001	1.01 (1.01-1.02)	<0.001
	Q1	Reference		Reference		Reference	
	Q2	0.93 (0.78-1.11)	0.431	0.91 (0.76-1.10)	0.333	0.96 (0.80-1.16)	0.694
	Q3	1.73 (1.42-2.10)	<0.001	1.54 (1.26-1.88)	<0.001	1.56 (1.27-1.91)	<0.001
	Q4	2.71 (2.18-3.37)	<0.001	2.21 (1.77-2.77)	<0.001	2.15 (1.67-2.78)	<0.001
	*P*_trend_		<0.001		<0.001		<0.001
	FT3/FT4	0.68 (0.63-0.72)	<0.001	0.73 (0.68-0.78)	<0.001	0.75 (0.69-0.81)	<0.001
	Q1	Reference		Reference		Reference	
	Q2	0.62 (0.49-0.78)	<0.001	0.68 (0.54-0.87)	0.002	0.70 (0.55-0.90)	0.005
	Q3	0.36 (0.29-0.45)	<0.001	0.43 (0.35-0.54)	<0.001	0.47 (0.36-0.61)	<0.001
	Q4	0.35 (0.29-0.44)	<0.001	0.44 (0.35-0.56)	<0.001	0.48 (0.37-0.62)	<0.001
	*P*_trend_		<0.001		<0.001		<0.001

^1^Model 1: adjusted for age and sex.

^2^Model 2: adjusted for age, sex, SBP, DBP, HbA1c, TC, TG, HDL-C, and LDL-C.

^3^Model 3: adjusted for age, sex, SBP, DBP, HbA1c, TC, TG, HDL-C, LDL-C, smoking, and drinking.

TFQI, thyroid feedback quantile-based index; PTFQI, parametric thyroid feedback quantile-based index; TSHI, TSH index; TT4RI, thyrotroph thyroxine resistance index; FT3/FT4, free triiodothyronine/free thyroxine; OR, odd ratio; CI, confidence interval; SBP, systolic blood pressure; DBP, diastolic blood pressure; HbA1c, glycated hemoglobin; TC, total cholesterol; TG, triglycerides; HDL-C, high-density lipoprotein cholesterol; LDL-C, low-density lipoprotein cholesterol; CHD, coronary heart disease.

The TSHI, TT4RI, TFQI, PTFQI and FT3/FT4 indices were divided into quartiles, Q1, TSHI < 0.53; Q2, 0.53 ≤ TSHI  ≤ 1.07; Q3, 1.07 ≤ TSHI  ≤  1.44; Q4, TSHI  > 1.44; Q1, TT4RI < 1.53; Q2, 1.53 ≤ TT4RI  ≤ 3.09; Q3, 3.09 ≤ TT4RI  ≤  24.25; Q4, TFQI  > 24.25; Q1, TFQI < -0.30; Q2, -0.30 ≤ TFQI  ≤ -0.01; Q3, -0.01 ≤ TFQI  ≤  0.29; Q4, TFQI  > 0.29; Q1, PTFQI < -0.34; Q2, -0.34 ≤ PTFQI  ≤ -0.06; Q3, -0.06 ≤ PTFQI  ≤  0.30; Q4, PTFQI  > 0.30; Q1, FT3/FT4 < 0.27; Q2, 0.27 ≤ FT3/FT4  ≤ 2.16; Q3, 2.16 ≤ FT3/FT4  ≤  2.63; Q4, FT3/FT4  > 2.63.

### Subgroup analyses for thyroid hormone sensitivity associated with risk of carotid plaque

In the subgroup analysis of sex and age, the association between thyroid hormone sensitivity and carotid plaque is shown in **Table 3** and **Table 4**. Regardless of sex, the association between thyroid hormone sensitivity and carotid plaque remained significant (*P* < 0.001). The OR values of thyroid hormone sensitivity and carotid plaque in males were higher than that in females. After multivariate adjustment, the association in middle-aged (≤ 60 years) patients was greater than in older (> 60 years) patients. Of all the indices representing central thyroid hormone sensitivity, the PTFQI had the highest OR value. The OR value of PTFQI in males (OR: 2.62; 95% CI: 2.04–3.35; *P* < 0.001) was higher than that in females (OR: 2.36; 95% CI: 1.94–2.87; *P* < 0.001), and the OR value of PTFQI in people ≤ 60 years old (OR: 3.23; 95% CI: 2.55–4.09; *P* < 0.001) was higher than that in people > 60 years old (OR: 2.16; 95% CI: 1.78–2.63; *P* < 0.001).

**Table 3:** Association between thyroid hormone sensitivity and carotid plaque according to sex.

Sex	Variables	Carotid plaque					
		OR (95% CI)^1^	*P-*value	OR (95% CI)^2^	*P-*value	OR (95% CI)^3^	*P-*value
Males	TFQI	2.26 (1.78-2.89)	< 0.001	1.85 (1.44-2.38)	< 0.001	1.77 (1.36-2.29)	< 0.001
	Q1	Reference		Reference		Reference	
	Q2	1.39 (1.08-1.79)	0.012	1.24 (0.95-1.61)	0.112	1.29 (0.98-1.68)	0.066
	Q3	1.92 (1.47-2.52)	< 0.001	1.66 (1.26-2.18)	< 0.001	1.61 (1.21-2.14)	0.001
	Q4	2.33 (1.76-3.07)	< 0.001	1.88 (1.41-2.50)	< 0.001	1.76 (1.31-2.37)	< 0.001
	PTFQI	2.62 (2.04-3.35)	< 0.001	2.12 (1.64-2.74)	< 0.001	2.00 (1.53-2.62)	< 0.001
	Q1	Reference		Reference		Reference	
	Q2	0.92 (0.71-1.19)	0.538	0.87 (0.67-1.13)	0.303	0.91 (0.70-1.20)	0.507
	Q3	2.02 (1.54-2.55)	< 0.001	1.70 (1.29-2.26)	< 0.001	1.68 (1.26-2.25)	< 0.001
	Q4	2.42 (1.84-3.18)	< 0.001	1.98 (1.49-2.63)	< 0.001	1.85 (1.38-2.48)	< 0.001
	TSHI	1.53 (1.32-1.76)	< 0.001	1.35 (1.17-1.57)	< 0.001	1.33 (1.14-1.55)	< 0.001
	Q1	Reference		Reference		Reference	
	Q2	1.10 (0.85-1.43)	0.453	1.00 (0.77-1.30)	0.983	1.01 (0.77-1.32)	0.928
	Q3	2.28 (1.74-2.97)	< 0.001	1.93 (1.46-2.54)	< 0.001	1.83 (1.38-2.44)	< 0.001
	Q4	1.80 (1.36-2.38)	< 0.001	1.46 (1.09-1.95)	0.010	1.44 (1.07-1.94)	0.017
	TT4RI	1.02 (1.01-1.03)	< 0.001	1.02 (1.01-1.02)	< 0.001	1.01 (1.01-1.02)	< 0.001
	Q1	Reference		Reference		Reference	
	Q2	0.91 (0.71-1.18)	0.490	0.86 (0.66-1.12)	0.272	0.92 (0.70-1.20)	0.527
	Q3	2.17 (1.65-2.86)	< 0.001	1.85 (1.40-2.46)	< 0.001	1.82 (1.37-2.43)	< 0.001
	Q4	2.44 (1.85-3.20)	< 0.001	2.00 (1.51-2.66)	< 0.001	1.90 (1.41-2.55)	< 0.001
	FT3/FT4	0.66 (0.61-0.72)	< 0.001	0.70 (0.65-0.77)	< 0.001	0.73 (0.67-0.80)	< 0.001
	Q1	Reference		Reference		Reference	
	Q2	0.66 (0.49-0.90)	0.008	0.71 (0.52-0.97)	0.031	0.71 (0.52-0.97)	0.034
	Q3	0.34 (0.25-0.46)	< 0.001	0.40 (0.29-0.54)	< 0.001	0.42 (0.31-0.59)	< 0.001
	Q4	0.29 (0.22-0.39)	< 0.001	0.36 (0.27-0.48)	< 0.001	0.39 (0.29-0.53)	< 0.001
Females	TFQI	1.97 (1.63-2.38)	< 0.001	1.59 (1.30-1.95)	< 0.001	1.33 (1.05-1.68)	< 0.001
	Q1	Reference		Reference		Reference	
	Q2	0.96 (0.78-1.18)	0.685	0.90 (0.73-1.11)	0.308	0.89 (0.72-1.10)	0.266
	Q3	1.30 (1.06-1.60)	0.013	1.19 (0.96-1.47)	0.112	1.13 (0.91-1.40)	0.266
	Q4	1.92 (1.55-2.38)	< 0.001	1.53 (1.22-1.92)	< 0.001	1.26 (0.97-1.64)	0.084
	PTFQI	2.36 (1.94-2.87)	< 0.001	1.87 (1.52-2.31)	< 0.001	1.66 (1.25-2.19)	< 0.001
	Q1	Reference		Reference		Reference	
	Q2	0.85 (0.70-1.04)	0.108	0.85 (0.69-1.04)	0.105	0.86 (0.70-1.06)	0.148
	Q3	1.36 (1.10-1.67)	0.005	1.24 (1.00-1.54)	0.054	1.24 (0.99-1.56)	0.058
	Q4	2.33 (1.86-2.93)	< 0.001	1.85 (1.46-2.35)	< 0.001	1.83 (1.32-2.54)	< 0.001
	TSHI	1.38 (1.24-1.53)	< 0.001	1.26 (1.13-1.41)	< 0.001	1.15 (1.02-1.30)	< 0.001
	Q1	Reference		Reference		Reference	
	Q2	0.92 (0.75-1.12)	0.403	0.90 (0.73-1.10)	0.297	0.90 (0.73-1.11)	0.315
	Q3	1.44 (1.16-1.79)	0.001	1.24 (0.99-1.56)	0.057	1.13 (0.89-1.43)	0.302
	Q4	1.80 (1.46-2.22)	< 0.001	1.52 (1.22-1.89)	< 0.001	1.32 (1.04-1.68)	0.024
	TT4RI	1.01 (1.01-1.01)	< 0.001	1.01 (1.00-1.01)	< 0.001	1.00 (1.00-1.01)	0.156
	Q1	Reference		Reference		Reference	
	Q2	0.80 (0.65-0.97)	0.025	0.79 (0.64-0.96)	0.020	0.80 (0.66-0.99)	0.035
	Q3	1.38 (1.11-1.70)	0.003	1.24 (1.00-1.55)	0.052	1.25 (1.00-1.57)	0.052
	Q4	2.23 (1.78-2.80)	< 0.001	1.77 (1.39-2.24)	< 0.001	1.74 (1.26-2.40)	0.001
	FT3/FT4	0.70 (0.65-0.75)	< 0.001	0.76 (0.71-0.82)	< 0.001	0.73 (0.65-0.83)	< 0.001
	Q1	Reference		Reference		Reference	
	Q2	0.70 (0.55-0.88)	0.002	0.82 (0.65-1.05)	0.116	0.91 (0.68-1.21)	0.514
	Q3	0.42 (0.34-0.52)	< 0.001	0.53 (0.42-0.66)	< 0.001	0.62 (0.45-0.86)	0.005
	Q4	0.45 (0.36-0.56)	< 0.001	0.59 (0.46-0.75)	< 0.001	0.69 (0.49-0.96)	0.026

^1^Model 1: adjusted for age;

^2^Model 2: adjusted for age, SBP, DBP, HbA1c, TC, TG, HDL-C, LDL-C;

^3^Model 3: adjusted for age, SBP, DBP, HbA1c, TC, TG, HDL-C, LDL-C, smoking, drinking.

OR, odds ratio; CI, confidence interval; TFQI, thyroid feedback quantile-based index; SBP, systolic blood pressure; DBP, diastolic blood pressure; HbA1c, glycated hemoglobin; TC, total cholesterol; TG, triglycerides; HDL-C, high-density lipoprotein cholesterol; LDL-C, low-density lipoprotein cholestero.

**Table 4:** Association between thyroid hormone sensitivity and carotid plaque according to age.

Age	Variables	Carotid plaque					
		OR (95% CI)^1^	*P-*value	OR (95% CI)^2^	*P-*value	OR (95% CI)^3^	*P-*value
≤60	TFQI	2.66 (2.11-3.36)	< 0.001	2.00 (1.56-2.55)	< 0.001	1.93 (1.48-2.50)	< 0.001
	Q1	Reference		Reference		Reference	
	Q2	1.44 (1.12-1.85)	0.004	1.32 (1.02-1.71)	0.034	1.38 (1.06-1.79)	0.017
	Q3	1.76 (1.37-2.27)	< 0.001	1.50 (1.15-1.95)	0.003	1.49 (1.14-1.94)	0.004
	Q4	2.78 (2.14-3.62)	< 0.001	2.06 (1.57-2.72)	< 0.001	1.97 (1.47-2.65)	< 0.001
	PTFQI	3.23 (2.55-4.09)	< 0.001	2.45 (1.91-3.15)	< 0.001	2.48 (1.88-3.28)	< 0.001
	Q1	Reference		Reference		Reference	
	Q2	0.95 (0.74-1.23)	0.714	0.92 (0.71-1.19)	0.537	0.95 (0.74-1.23)	0.709
	Q3	1.85 (1.42-2.40)	< 0.001	1.49 (1.13-1.95)	0.004	1.51 (1.15-2.00)	0.003
	Q4	3.11 (2.39-4.03)	< 0.001	2.41 (1.84-3.17)	< 0.001	2.53 (1.87-3.43)	< 0.001
	TSHI	1.70 (1.47-1.95)	< 0.001	1.47 (1.27-1.70)	< 0.001	1.42 (1.22-1.66)	< 0.001
	Q1	Reference		Reference		Reference	
	Q2	1.04 (0.81-1.34)	0.739	0.95 (0.74-1.24)	0.720	0.98 (0.75-1.27)	0.861
	Q3	2.18 (1.69-2.81)	< 0.001	1.83 (1.40-2.38)	< 0.001	1.81 (1.38-2.38)	< 0.001
	Q4	2.42 (1.86-3.15)	< 0.001	1.83 (1.39-2.41)	< 0.001	1.78 (1.33-2.39)	< 0.001
	TT4RI	1.02 (1.02-1.03)	< 0.001	1.02 (1.01-1.02)	< 0.001	1.02 (1.01-1.02)	< 0.001
	Q1	Reference		Reference		Reference	
	Q2	0.91 (0.71-1.17)	0.467	0.87 (0.67-1.12)	0.278	0.90 (0.69-1.16)	0.411
	Q3	2.04 (1.57-2.65)	< 0.001	1.64 (1.25-2.16)	< 0.001	1.68 (1.27-2.23)	< 0.001
	Q4	3.05 (2.35-3.97)	< 0.001	2.36 (1.79-3.10)	< 0.001	2.50 (1.85-3.39)	< 0.001
	FT3/FT4	0.64 (0.59-0.69)	< 0.001	0.71 (0.65-0.77)	< 0.001	0.70 (0.63-0.77)	< 0.001
	Q1	Reference		Reference		Reference	
	Q2	0.67 (0.51-0.88)	0.004	0.76 (0.57-1.01)	0.056	0.74 (0.55-1.00)	0.050
	Q3	0.32 (0.25-0.42)	<0.001	0.41 (0.31-0.55)	<0.001	0.41 (0.30-0.56)	<0.001
	Q4	0.30 (0.23-0.39)	<0.001	0.42 (0.32-0.56)	<0.001	0.42 (0.30-0.57)	<0.001
>60	TFQI	1.87 (1.54-2.26)	< 0.001	1.65 (1.35-2.01)	< 0.001	1.38 (1.10-1.72)	< 0.001
	Q1	Reference		Reference		Reference	
	Q2	1.00 (0.82-1.22)	0.992	0.93 (0.76-1.14)	0.456	0.91 (0.74-1.12)	0.362
	Q3	1.46 (1.18-1.80)	< 0.001	1.37 (1.11-1.70)	0.004	1.29 (1.04-1.61)	0.021
	Q4	1.85 (1.49-2.31)	< 0.001	1.61 (1.28-2.02)	< 0.001	1.30 (1.01-1.67)	0.044
	PTFQI	2.16 (1.78-2.63)	< 0.001	1.85 (1.50-2.27)	< 0.001	1.51 (1.18-1.94)	< 0.001
	Q1	Reference		Reference		Reference	
	Q2	0.88 (0.72-1.07)	0.202	0.87 (0.71-1.06)	0.165	0.90 (0.73-1.10)	0.294
	Q3	1.52 (1.23-1.87)	< 0.001	1.42 (1.15-1.76)	0.001	1.36 (1.10-1.69)	0.006
	Q4	2.06 (1.64-2.59)	< 0.001	1.75 (1.38-2.22)	< 0.001	1.38 (1.04-1.83)	0.027
	TSHI	1.34 (1.21-1.49)	< 0.001	1.26 (1.13-1.40)	< 0.001	1.16 (1.04-1.31)	< 0.001
	Q1	Reference		Reference		Reference	
	Q2	1.01 (0.83-1.23)	0.944	0.98 (0.80-1.20)	0.817	0.98 (0.80-1.20)	0.840
	Q3	1.55 (1.25-1.92)	< 0.001	1.37 (1.10-1.71)	0.005	1.21 (0.97-1.53)	0.097
	Q4	1.65 (1.33-2.03)	< 0.001	1.48 (1.19-1.84)	< 0.001	1.28 (1.01-1.61)	0.038
	TT4RI	1.01 (1.00-1.01)	< 0.001	1.01 (1.00-1.01)	0.003	1.00 (1.00-1.00)	0.454
	Q1	Reference		Reference		Reference	
	Q2	0.86 (0.70-1.04)	0.119	0.84 (0.69-1.03)	0.090	0.88 (0.72-1.07)	0.202
	Q3	1.53 (1.24-1.88)	< 0.001	1.43 (1.16-1.77)	0.001	1.38 (1.11-1.72)	0.004
	Q4	2.05 (1.63-2.57)	< 0.001	1.74 (1.37-2.21)	< 0.001	1.39 (1.05-1.85)	0.020
	FT3/FT4	0.70 (0.66-0.75)	< 0.001	0.74 (0.69-0.80)	< 0.001	0.78 (0.71-0.86)	< 0.001
	Q1	Reference		Reference		Reference	
	Q2	0.66 (0.52-0.84)	0.001	0.74 (0.58-0.95)	0.017	0.83 (0.63-1.09)	0.180
	Q3	0.41 (0.32-0.51)	<0.001	0.46 (0.36-0.59)	<0.001	0.56 (0.42-0.75)	<0.001
	Q4	0.40 (0.32-0.50)	<0.001	0.47 (0.37-0.59)	<0.001	0.57 (0.42-0.76)	<0.001

^1^Model 1: adjusted for sex;

^2^Model 2: adjusted for sex, SBP, DBP, HbA1c, TC, TG, HDL-C, LDL-C;

^3^Model 3: adjusted for sex, SBP, DBP, HbA1c, TC, TG, HDL-C, LDL-C, smoking, drinking.

OR, odds ratio; CI, confidence interval; TFQI, thyroid feedback quantile-based index; SBP, systolic blood pressure; DBP, diastolic blood pressure; HbA1c, glycated hemoglobin; TC, total cholesterol; TG, triglycerides; HDL-C, high-density lipoprotein cholesterol; LDL-C, low-density lipoprotein cholesterol.

As shown in **Tables 5** and **Table 6**, after multi-adjustment, the OR values of central thyroid hormone sensitivity of smokers were higher than that of non-smokers, and the OR value of peripheral thyroid hormone sensitivity was lower than that of non-smokers. Unlike non-drinkers, drinkers had higher OR values for TFQI, PTFQI, and TSHI and lower OR values for FT3/FT4. The OR value of PTFQI was the highest of central thyroid hormone sensitivity. The OR value of PTFQI in smokers (OR: 2.00; 95% CI: 1.51–2.65; *P* < 0.001) was higher than that in non-smokers (OR: 1.54; 95% CI: 1.17–2.03; *P =* 0.002), and the OR value of PTFQI in drinkers (OR: 2.02; 95% CI: 1.54–2.64; *P* < 0.001) was higher than that in non-drinkers (OR: 1.61; 95% CI: 1.20–2.14; *P =* 0.001).

**Table 5:** Association between thyroid hormone sensitivity and carotid plaque according to smoking.

Group	Variables	Carotid plaque					
		OR (95% CI)^1^	*P-*value	OR (95% CI)^2^	*P-*value	OR (95% CI)^3^	*P-*value
Smoking	TFQI	2.20 (1.69-2.87)	< 0.001	1.81 (1.37-2.37)	< 0.001	1.76 (1.34-2.32)	< 0.001
	Q1	Reference		Reference		Reference	
	Q2	0.96 (0.79-1.17)	0.682	0.90 (0.74-1.10)	0.309	0.87 (0.71-1.06)	0.169
	Q3	1.31 (1.08-1.60)	0.007	1.21 (0.98-1.48)	0.074	1.11 (0.90-1.37)	0.322
	Q4	2.04 (1.66-2.52)	< 0.001	1.65 (1.33-2.06)	< 0.001	1.26 (0.97-1.63)	0.079
	PTFQI	2.49 (1.91-3.25)	< 0.001	2.04 (1.55-2.70)	< 0.001	2.00 (1.51-2.65)	< 0.001
	Q1	Reference		Reference		Reference	
	Q2	0.87 (0.72-1.05)	0.153	0.86 (0.71-1.05)	0.128	0.86 (0.71-1.04)	0.122
	Q3	1.33 (1.08-1.62)	0.006	1.22 (0.99-1.50)	0.062	1.17 (0.94-1.45)	0.164
	Q4	2.37 (1.90-2.96)	< 0.001	1.92 (1.53-2.42)	< 0.001	1.65 (1.20-2.27)	0.002
	TSHI	1.49 (1.23-1.73)	< 0.001	1.35 (1.15-1.57)	< 0.001	1.33 (1.13-1.55)	< 0.001
	Q1	Reference		Reference		Reference	
	Q2	0.93 (0.77-1.13)	0.477	0.90 (0.74-1.10)	0.286	0.88 (0.72-1.07)	0.196
	Q3	1.47 (1.19-1.81)	< 0.001	1.31 (1.06-1.62)	0.014	1.12 (0.89-1.41)	0.329
	Q4	1.80 (1.47-2.20)	< 0.001	1.52 (1.23-1.88)	< 0.001	1.22 (0.97-1.54)	0.094
	TT4RI	1.01 (1.01-1.02)	< 0.001	1.01 (1.00-1.01)	0.007	1.01 (1.00-1.01)	0.011
	Q1	Reference		Reference		Reference	
	Q2	0.83 (0.69-1.01)	0.061	0.82 (0.67-0.99)	0.041	0.82 (0.67-1.00)	0.045
	Q3	1.36 (1.11-1.66)	0.003	1.23 (1.00-1.52)	0.051	1.19 (0.96-1.48)	0.115
	Q4	2.34 (1.87-2.91)	< 0.001	1.88 (1.49-2.37)	< 0.001	1.65 (1.21-2.26)	0.002
	FT3/FT4	0.66 (0.60-0.72)	< 0.001	0.71 (0.64-0.78)	< 0.001	0.71 (0.65-0.78)	< 0.001
	Q1	Reference		Reference		Reference	
	Q2	0.67 (0.53-0.84)	< 0.001	0.77 (0.61-0.97)	0.029	0.91 (0.69-1.21)	0.532
	Q3	0.41 (0.34-0.51)	<0.001	0.51 (0.41-0.63)	<0.001	0.66 (0.48-0.92)	0.014
	Q4	0.43 (0.35-0.54)	<0.001	0.56 (0.44-0.70)	<0.001	0.73 (0.52-1.01)	0.057
Non-smoking	TFQI	2.02 (1.68-2.43)	< 0.001	1.66 (1.37-2.02)	< 0.001	1.28 (1.02-1.61)	0.035
	Q1	Reference		Reference		Reference	
	Q2	1.62 (1.22-2.16)	0.001	1.45 (1.08-1.95)	0.015	1.45 (1.07-1.95)	0.015
	Q3	2.02 (1.51-2.72)	< 0.001	1.73 (1.28-2.34)	< 0.001	1.70 (1.26-2.30)	0.001
	Q4	2.10 (1.56-2.82)	< 0.001	1.71 (1.26-2.32)	0.001	1.67 (1.22-2.26)	0.001
	PTFQI	2.41 (1.99-2.92)	< 0.001	1.95 (1.59-2.38)	< 0.001	1.54 (1.17-2.03)	0.002
	Q1	Reference		Reference		Reference	
	Q2	0.97 (0.73-1.29)	0.825	0.93 (0.69-1.24)	0.611	0.92 (0.69-1.23)	0.579
	Q3	2.37 (1.74-3.22)	< 0.001	2.00 (1.46-2.74)	< 0.001	1.97 (1.44-2.71)	< 0.001
	Q4	2.31 (1.72-3.09)	< 0.001	1.91 (1.41-2.58)	< 0.001	1.86 (1.37-2.53)	< 0.001
	TSHI	1.41 (1.27-1.57)	< 0.001	1.29 (1.16-1.44)	< 0.001	1.14 (1.01-1.28)	0.036
	Q1	Reference		Reference		Reference	
	Q2	1.18 (0.89-1.568)	0.253	1.10 (0.82-1.48)	0.514	1.09 (0.82-1.46)	0.555
	Q3	2.32 (1.73-3.11)	< 0.001	1.92 (1.42-2.60)	< 0.001	1.89 (1.39-2.55)	< 0.001
	Q4	1.93 (1.42-2.62)	< 0.001	1.61 (1.18-2.21)	< 0.001	1.57 (1.15-2.16)	0.005
	TT4RI	1.01 (1.01-1.02)	< 0.001	1.01 (1.00-1.01)	< 0.001	1.00 (1.00-1.01)	0.117
	Q1	Reference		Reference		Reference	
	Q2	0.94 (0.70-1.25)	0.653	0.89 (0.67-1.20)	0.454	0.89 (0.67-1.20)	0.441
	Q3	2.52 (1.85-3.44)	< 0.001	2.15 (1.57-2.96)	< 0.001	2.12 (1.54-2.92)	< 0.001
	Q4	2.24 (1.67-2.99)	< 0.001	1.86 (1.37-2.51)	< 0.001	1.82 (1.34-2.47)	< 0.001
	FT3/FT4	0.71 (0.67-0.76)	< 0.001	0.77 (0.72-0.83)	< 0.001	0.79 (0.70-0.89)	< 0.001
	Q1	Reference		Reference		Reference	
	Q2	0.72 (0.52-1.00)	0.048	0.76 (0.55-1.07)	0.111	0.77 (0.55-1.07)	0.117
	Q3	0.37 (0.27-0.51)	<0.001	0.42 (0.30-0.59)	<0.001	0.43 (0.30-0.60)	<0.001
	Q4	0.32 (0.23-0.43)	<0.001	0.39 (0.28-0.54)	<0.001	0.39 (0.29-0.54)	<0.001

^1^Model 1: adjusted for age and sex;

^2^Model 2: adjusted for age, sex, SBP, DBP, HbA1c, TC, TG, HDL-C, LDL-C;

^3^Model 3: adjusted for age, sex, SBP, DBP, HbA1c, TC, TG, HDL-C, LDL-C, drinking.

OR, odd ratio; CI, confidence interval; SBP, systolic blood pressure; DBP, diastolic blood pressure; HbA1c, glycated hemoglobin; TC, total cholesterol; TG, triglycerides; HDL-C, high-density lipoprotein cholesterol; LDL-C, low-density lipoprotein cholesterol.

**Table 6:** Association between thyroid hormone sensitivity and carotid plaque according to drinking.

Group	Variables	Carotid plaque					
		OR (95% CI)^1^	*P-*value	OR (95% CI)^2^	*P-*value	OR (95% CI)^3^	*P-*value
Drinking	TFQI	1.81 (1.42-2.32)	< 0.001	1.57 (1.22-2.02)	< 0.001	1.72 (1.33-2.23)	< 0.001
	Q1	Reference		Reference		Reference	
	Q2	0.92 (0.77-1.12)	0.422	0.90 (0.74-1.09)	0.265	0.91 (0.75-1.10)	0.313
	Q3	1.13 (0.93-1.38)	0.216	1.09 (0.88-1.33)	0.423	1.08 (0.88-1.32)	0.479
	Q4	1.94 (1.44-2.60)	< 0.001	1.68 (1.24-2.26)	0.001	1.48 (1.09-2.00)	0.013
	PTFQI	2.12 (1.65-2.74)	< 0.001	1.79 (1.37-2.32)	< 0.001	2.02 (1.54-2.64)	< 0.001
	Q1	Reference		Reference		Reference	
	Q2	0.89 (0.74-1.06)	0.196	0.88 (0.73-1.06)	0.172	0.91 (0.75-1.09)	0.308
	Q3	1.21 (0.99-1.49)	0.061	1.16 (0.94-1.42)	0.172	1.15 (0.93-1.42)	0.186
	Q4	4.20 (2.62-6.73)	< 0.001	3.37 (2.09-5.44)	< 0.001	2.49 (1.52-4.08)	< 0.001
	TSHI	1.41 (1.21-1.65)	< 0.001	1.29 (1.10-1.51)	0.002	1.35 (1.15-1.59)	< 0.001
	Q1	Reference		Reference		Reference	
	Q2	0.92 (0.77-1.13)	0.477	0.94 (0.77-1.13)	0.490	0.96 (0.79-1.16)	0.678
	Q3	1.47 (1.19-1.81)	< 0.001	1.13 (0.90-1.42)	0.286	1.09 (0.86-1.37)	0.482
	Q4	1.80 (1.47-2.20)	< 0.001	1.31 (1.02-1.68)	0.032	1.25 (0.98-1.61)	0.076
	TT4RI	1.00 (1.00-1.01)	0.029	1.00 (1.00-1.01)	0.137	1.00 (1.00-1.01)	0.072
	Q1	Reference		Reference		Reference	
	Q2	0.88 (0.74-1.05)	0.166	0.86 (0.72-1.03)	0.109	0.89 (0.74-1.07)	0.223
	Q3	1.28 (1.04-1.58)	0.018	1.22 (0.99-1.51)	0.063	1.22 (0.99-1.51)	0.063
	Q4	3.95 (2.51-6.23)	< 0.001	3.19 (2.01-5.06)	< 0.001	2.39 (1.49-3.85)	< 0.001
	FT3/FT4	0.67 (0.62-0.74)	< 0.001	0.72 (0.66-0.79)	< 0.001	0.68 (0.62-0.75)	< 0.001
	Q1	Reference		Reference		Reference	
	Q2	0.31 (0.19-0.51)	< 0.001	0.38 (0.23-0.62)	< 0.001	0.53 (0.32-0.88)	0.013
	Q3	0.23 (0.15-0.37)	<0.001	0.29 (0.18-0.46)	<0.001	0.42 (0.26-0.70)	0.001
	Q4	0.24 (0.15-0.37)	<0.001	0.31 (0.19-0.50)	<0.001	0.45 (0.27-0.74)	0.002
Non-drinking	TFQI	1.63 (1.30-2.05)	< 0.001	1.43 (1.13-1.82)	0.003	1.32 (1.04-1.68)	0.024
	Q1	Reference		Reference		Reference	
	Q2	1.64 (1.21-2.23)	0.002	1.39 (1.02-1.91)	0.039	1.48 (1.08-2.04)	0.015
	Q3	2.20 (1.63-2.97)	< 0.001	1.87 (1.38-2.54)	< 0.001	2.00 (1.47-2.73)	< 0.001
	Q4	2.00 (1.51-2.64)	< 0.001	1.65 (1.23-2.20)	0.001	1.84 (1.37-2.47)	< 0.001
	PTFQI	2.12 (1.63-2.77)	< 0.001	1.84 (1.40-2.43)	< 0.001	1.61 (1.20-2.14)	0.001
	Q1	Reference		Reference		Reference	
	Q2	0.81 (0.59-1.12)	0.203	0.77 (0.55-1.06)	0.112	0.79 (0.57-1.10)	0.160
	Q3	2.14 (1.57-2.91)	< 0.001	1.77 (1.29-2.42)	< 0.001	1.94 (1.41-2.68)	< 0.001
	Q4	2.08 (1.56-2.77)	< 0.001	1.72 (1.28-2.32)	< 0.001	1.96 (1.45-2.66)	< 0.001
	TSHI	1.21 (1.08-1.35)	0.001	1.17 (1.04-1.31)	0.010	1.14 (1.02-1.29)	0.027
	Q1	Reference		Reference		Reference	
	Q2	1.06 (0.78-1.44)	0.726	0.92 (0.67-1.27)	0.617	0.96 (0.70-1.32)	0.801
	Q3	2.11 (1.57-2.82)	< 0.001	1.78 (1.32-2.40)	< 0.001	1.95 (1.44-2.65)	< 0.001
	Q4	1.89 (1.40-2.54)	< 0.001	1.56 (1.15-2.12)	0.004	1.75 (1.29-2.39)	< 0.001
	TT4RI	1.03 (1.02-1.04)	< 0.001	1.03 (1.02-1.04)	< 0.001	1.02 (1.01-1.03)	0.001
	Q1	Reference		Reference		Reference	
	Q2	0.71 (0.51-0.98)	0.036	0.67 (0.48-0.94)	0.020	0.68 (0.49-0.95)	0.024
	Q3	2.11 (1.56-2.86)	< 0.001	1.74 (1.28-2.37)	< 0.001	1.88 (1.37-2.57)	< 0.001
	Q4	1.98 (1.50-2.63)	< 0.001	1.64 (1.23-2.19)	0.001	1.84 (1.37-2.48)	< 0.001
	FT3/FT4	0.68 (0.61-0.75)	< 0.001	0.73 (0.66-0.81)	< 0.001	0.80 (0.71-0.90)	< 0.001
	Q1	Reference		Reference		Reference	
	Q2	0.97 (0.76-1.23)	0.792	1.00 (0.78-1.28)	0.994	0.96 (0.75-1.23)	0.741
	Q3	0.40 (0.29-0.55)	<0.001	0.46 (0.33-0.64)	<0.001	0.40 (0.29-0.56)	<0.001
	Q4	0.38 (0.28-0.51)	<0.001	0.45 (0.33-0.61)	<0.001	0.40 (0.29-0.54)	<0.001

^1^Model 1: adjusted for age and sex;

^2^Model 2: adjusted for age, sex, SBP, DBP, HbA1c, TC, TG, HDL-C, LDL-C;

^3^Model 3: adjusted for age, sex, SBP, DBP, HbA1c, TC, TG, HDL-C, LDL-C, smoking.

OR, odd ratio; CI, confidence interval; SBP, systolic blood pressure; DBP, diastolic blood pressure; HbA1c, glycated hemoglobin; TC, total cholesterol; TG, triglycerides; HDL-C, high-density lipoprotein cholesterol; LDL-C, low-density lipoprotein cholesterol.

## Discussion

To the best of our knowledge, this is the first study to evaluate the association between central and peripheral thyroid hormone sensitivity and the risk of carotid plaque in a large sample of patients with CHD in China. In this cross-sectional study, we found that the central thyroid hormone sensitivity indices TFQI, PTFQI, TSHI, and TT4RI were positively associated with the risk of carotid plaque in patients with CHD. With a gradual increase in TSHI, TT4RI, TFQI, and PTFQI, the OR value of carotid plaque also gradually increased. Peripheral thyroid hormone sensitivity index FT3/FT4 was negatively associated with the risk of carotid plaque, and the OR value of carotid plaque gradually decreased as FT3/FT4 gradually increased. Furthermore, the association remained consistent when stratified for sex, age, and smoking and drinking status.

Previous studies have shown that carotid plaque can predicte cardiovascular events and improved risk prediction for CHD (31). Due to the various changes of thyroid hormones in patients with CHD (32), the various effects of thyroid hormones in patients with CHD (33) and carotid plaque deserve attention (34). However, previous studies showed that higher TSH (11), lower TSH (35), lower FT3 (34), higher FT3, and higher FT4 (10) levels were all associated with the risk of carotid plaque. Inconsistent findings from previous studies have highlighted that TSH or thyroid hormone levels alone may not be sufficient to explain the association between the thyroid system and carotid plaque. Given these inconsistencies in previously proposed central thyroid hormone sensitivity indices (TSHI, TT4RI) and peripheral thyroid hormone sensitivity indices FT3/FT4 (27, 28), in 2019, Laclaustra et al. (29) proposed new resistance indices of central thyroid hormones, TFQI and PTFQI. TFQI is based on the empirical joint distribution of FT4 and TSH with the advantage of not yielding extreme values in cases of thyroid dysfunction. PTFQI is an index that can be calculated for any new value or adapted to other populations, with the same range and interpretation as an approximation. These new indices may have smaller deviations and can systematically reflect the regulation of thyroid hormone homeostasis compared to a single index, which can better explain the different associations between the changes in thyroid hormones and carotid plaque.

Many studies have reported an association between thyroid hormone levels and carotid plaque formation (36, 37). A cross-sectional study found that TSH was independently associated with carotid plaque, especially in participants with elevated TSH levels (11). A prospective cohort study in a Chinese population reported that higher mean levels and higher values of changes in FT3 and FT4 were associated with a higher risk of carotid atherosclerosis in euthyroid middle-aged and older participants (10). More recently, alterations in thyroid function, even within the reference ranges, were also found to be associated with atherosclerosis in the general population and patients with angina pectoris (38). Unlike this study, they did not calculate the association between central or peripheral thyroid hormone sensitivity and carotid plaque. this study showed that elevated TSHI, TT4RI, TFQI, and PTFQI calculated based on TSH and FT4 were associated with increased risk of carotid plaque, and elevated FT3/FT4 was associated with decreased risk of carotid plaque. Among central thyroid hormone sensitivity indices, PTFQI had the best sensitivity. In addition, when used as a reference in the Q1 group, central thyroid hormone sensitivity indices TSHI, TT4RI, TFQI and PTFQI were positively associated with carotid plaque in the Q4 group, and the sensitivity was the strongest in the Q4 group. Interestingly, our study also found that when used as a reference in the Q1 group, peripheral thyroid hormone sensitivity index FT3/FT4 was negatively associated with carotid plaque in the Q4 group, and the sensitivity was the strongest in the Q4 group. As many studies confirmed the positive effects of elevated FT3/FT4 on cardiovascular risk and arterial stiffness markers (39–41). However, studies have reported conflicting views. A cross-sectional analysis by Dörr et al. (35) reported an association between subclinical hyperthyroidism and carotid plaque, with an OR value of 1.67, suggesting that participants with reduced TSH levels should undergo regular screening for atherosclerotic risk factors and early treatment. A cross-sectional study of Chinese participants with type 2 diabetes and euthyroidism showed that low-normal FT3 levels were associated with a high prevalence of atherosclerosis (34). Differences in the methodologies used in various studies may also partly explain the contrasting results: differences in carotid plaque measurements, sample size, study population, differences in the normal range of the thyroid hormones, and differences in the definition of thyroid function are possible causes of the contrasting results.

To address sex- and age-specific differences noted in previous studies (42, 43), this study analyzed the association between thyroid hormone sensitivity and diabetes by sex and age, respectively. The risk of carotid plaque in patients with CHD is higher in males than in females and continues to increase with age (5, 13). The same sex-stratification was seen in this study, but contrary to most studies, our results show that, regardless of age, decreased central thyroid hormone sensitivity is associated with carotid plaques and was more strongly associated with people ≤ 60 years. This may be because this study falls within the CHD population, in which, the average age is > 60, belongs to the middle-aged and older population, and has a partial age bias.

Non-smoking and non-drinking are both related to a reduced risk of atherosclerosis. The Wisconsin Smokers’ Health Study, which examined the relationship between smoking burden and carotid plaque, showed that smoking cessation was related to slower progress of carotid plaque (44). Smoking is related to an increased cardiovascular risk, and smokers should be targeted for atherosclerosis prevention (45). Drinking is related to carotid plaque formation, especially excessive drinking (46, 47). In this study, decreased central thyroid hormone sensitivity is related to an increased risk of carotid plaque in CHD patients, especially among smokers and drinkers.

Carotid plaque and CIMT are markers of carotid atherosclerosis (48, 49). More and more studies have analyzed the possible pathways of the thyroid hormone’s effect on atherosclerosis. A previous study reported that type 2 iodothyronine deiodinase (D2), a thyroid hormone-activating enzyme that converts T4 to T3, was expressed in arterial smooth muscle cells (hCASMCs) (50). Activation of intracellular thyroid hormone by D2 inhibits the DNA synthesis and migration activity of hCASMCs (51), suggesting that thyroid hormone has a direct inhibitory effect on atherosclerosis. An excessively low thyroid hormone concentration can reduce the production of reactive oxygen species and the degree of vasodilation, indicating effects on endothelial cells (52, 53). Furthermore, thyroid hormone induces rapid activation of phosphoinositide 3-kinase (PI3K)/Akt/endothelial nitric oxide synthase in endothelial cells. We recently reported that the transformation of T4 to T3 by D2 is involved in thyroid receptor α1/PI3K-mediated non-genomic effects of T4 in human umbilical vein endothelial cells, including stimulation of Akt phosphorylation and Rac activation (54). These findings suggest that thyroid hormone induces endothelial cell migration, which is important for vascular repair and angiogenesis against the progression of atherosclerosis. In addition, TSH itself stimulates the proliferation of vascular smooth muscle cells, thereby promoting atherosclerosis (55). In this study, thyroid hormone sensitivity was significantly associated with carotid plaque, especially at high hormone levels, suggesting that the direct effects of thyroid hormones on the arterial wall may be involved in the progression of atherosclerosis.

From a clinical point of view, there seems to be a gradient of increasing thyroid hormones levels when carotid plaque formation in patients with CHD. As resistance to thyroid hormone is one of the differential diagnoses when both FT4 and TSH are elevated (56), this results offer an explanation for thyroid profiles commonly found in patients with CHD who have carotid plaque. That is, at the population level, measurements of resistance to thyroid hormone are cross-sectionally associated with carotid plaque, and periodic screenings on thyroid hormones in patients with CHD were recommended to aid in early prevention of carotid plaque.

## Limitation

The strengths of this study include a large number of participants, detailed information on available covariates for adjustment analysis, and analysis of the new thyroid hormone sensitivity indices. This study also performed secondary analyses among euthyroid participants. However, this study has several limitations. First, ultrasound may not be as accurate as high-resolution magnetic resonance imaging or computed tomography for plaque assessment; however, ultrasound has certain advantages regarding safety and non-invasiveness. Second, as the data came from the Hospital Information System and self-reported information, sufficient body mass index data could not be obtained, and the specific amount of smoking and drinking could not be identified, which would result in underestimation for the effect. Third, The study was conducted in a non-randomized population of inpatients with CHD, which may have selection bias leading to under- or over-estimation of the observed associations. However, this study was multi-center, with strict inclusion and exclusion criteria, and the diagnosis of the disease was uniform and strict, and the results were reliable to some extent. Finally, the measurement methods may differ across laboratories in a multi-center study. However, practitioners assess the quality of external clinical laboratories at each center, and physicians keep records, greatly increasing their reliability.

## Conclusions

The increase in central thyroid hormone indices represents an decrease in central thyroid hormone sensitivity. This study showed that thyroid hormone sensitivity is significantly associated with carotid plaque in patients with CHD. This association is more significant in males, patients aged ≤ 60 years, smokers and drinkers. Evaluation of resistance to thyroid hormone may have important clinical significance for risk stratification and individualized treatment of patients with atherosclerosis.

## Data availability statement

The original contributions presented in the study are included in the article/**Supplementary Material**. Further inquiries can be directed to the corresponding authors.

## Ethics statement

This study was approved by the ethics committee of the Tianjin University of Traditional Chinese Medicine (approval number TJUTCM-EC20190008) and registered with the Chinese Clinical Trial Registry on July 14, 2019 (registration number ChiCTR-1900024535) and in ClinicalTrials.gov on July 18, 2019 (registration number NCT04026724). Written informed consent for participation was not required for this study in accordance with the national legislation and the institutional requirements.

## Author contributions

CY, RY, CL, and LL: review and editing. YL, ZL, and TY: writing original draft and analyzing the data. LY, FL, TN, and SG: participated in data collection. All the authors have read and approved the final manuscript.

## Funding

This study was supported by the National Basic Research Program of China (973 project, grant number 2014CB542902).

## Acknowledgments

We thank all the participants in the study, the members of the survey teams, and the groups for providing financial support.

## Conflict of interest

The authors declare that the research was conducted in the absence of any commercial or financial relationships that could be construed as a potential conflict of interest.

## Publisher’s note

All claims expressed in this article are solely those of the authors and do not necessarily represent those of their affiliated organizations, or those of the publisher, the editors and the reviewers. Any product that may be evaluated in this article, or claim that may be made by its manufacturer, is not guaranteed or endorsed by the publisher.

## Abbreviations

AITD, autoimmune thyroid disease; CHD, coronary heart disease; CI, confidence interval; CIMT, carotid intima-media thickness; CVD, cardiovascular disease; D2, type 2 iodothyronine deiodinase; DBP, diastolic blood pressure; FT3, free triiodothyronine; FT4, free thyroxine; FT3/FT4, free triiodothyronine/free thyroxine; HbA1c, glycated hemoglobin; hCASMC, human coronary arterial smooth muscle cell; HDL-C, high-density lipoprotein cholesterol; ICD-10, International Classification of Diseases 10th revision; LDL-C, low-density lipoprotein cholesterol; ORs, odd ratios; PI3K, phosphoinositide 3-kinase; PTFQI, parametric thyroid feedback quantile-based index; SBP, systolic blood pressure; TC, total cholesterol; TFQI, thyroid feedback quantile-based index; TG, triglyceride; TSH, thyroid-stimulating hormone; TSHI, TSH index; TT4RI, thyrotroph thyroxine resistance index.
