# Supplementary material for: Impaired sensitivity to thyroid hormones and carotid plaque in patients with coronary heart disease: A RCSCD-TCM study in China
Source: Front Endocrinol (Lausanne). 2022 Sep 27;13:940633. doi: 10.3389/fendo.2022.940633 (PMC9552887; doi:10.3389/fendo.2022.940633)
Supplement: Supplementary file 1 [file DataSheet_1.docx]

**Supplemental Materials**

**Table S1 Association between thyroid hormone sensitivity and carotid plaque in patients with CHD with non-autoimmune thyroid disease**

| **Variables** | **Carotid plaque (*n*=6,553)** | | | | | |
| --- | --- | --- | --- | --- | --- | --- |
|  | **OR (95% CI)^1^** | ***P*-value** | **OR (95% CI)^2^** | ***P*-value** | **OR (95% CI)^3^** | ***P*-value** |
| TFQI | 2.09(1.79-2.43) | <0.001 | 1.72(1.47-2.01) | <0.001 | 1.52(1.28-1.80) | <0.001 |
| PTFQI | 2.46(2.11-2.87) | <0.001 | 1.99(1.69-2.34) | <0.001 | 1.78(1.47-2.15) | <0.001 |
| TSHI | 1.44(1.32-1.57) | <0.001 | 1.31(1.19-1.43) | <0.001 | 1.23(1.11-1.35) | <0.001 |
| TT4RI | 1.01(1.01-1.02) | <0.001 | 1.01(1.01-1.01) | <0.001 | 1.01(1.00-1.01) | 0.003 |
| FT3/FT4 | 0.68(0.65-0.72) | <0.001 | 0.73(0.69-0.78) | <0.001 | 0.75(0.70-0.81) | <0.001 |

^1^Model 1: adjusted for age and sex

^2^Model 2: adjusted for age, sex, SBP, DBP, HbA1c, TC, TG, HDL-C, and LDL-C

^3^Model 3: adjusted for age, sex, SBP, DBP, HbA1c, TC, TG, HDL-C, LDL-C, smoking, and drinking

TFQI, thyroid feedback quantile-based index; PTFQI, parametric thyroid feedback quantile-based index; TSHI, TSH index; TT4RI, thyrotroph thyroxine resistance index; FT3/FT4, free triiodothyronine/ free thyroxine; OR, odd ratio; CI, confidence interval; SBP, systolic blood pressure; DBP, diastolic blood pressure; HbA1c, glycated hemoglobin; TC, total cholesterol; TG, triglycerides; HDL-C, high-density lipoprotein cholesterol; LDL-C, low-density lipoprotein cholesterol.

**Table S2 Association between thyroid hormone sensitivity and carotid plaque in patients with CHD with non-rheumatological disease**

| **Variables** | **Carotid plaque (*n*=6,454)** | | | | | |
| --- | --- | --- | --- | --- | --- | --- |
|  | **OR (95% CI)^1^** | ***P*-value** | **OR (95% CI)^2^** | ***P*-value** | **OR (95% CI)^3^** | ***P*-value** |
| TFQI | 2.07(1.78-2.41) | <0.001 | 1.70(1.45-1.99) | <0.001 | 1.49(1.25-1.78) | <0.001 |
| PTFQI | 2.44(2.09-2.85) | <0.001 | 1.97(1.67-2.32) | <0.001 | 1.75(1.45-2.12) | <0.001 |
| TSHI | 1.42(1.30-1.55) | <0.001 | 1.29(1.18-1.41) | <0.001 | 1.21(1.10-1.33) | <0.001 |
| TT4RI | 1.01(1.01-1.02) | <0.001 | 1.01(1.01-1.01) | <0.001 | 1.00(1.00-1.01) | 0.003 |
| FT3/FT4 | 0.68(0.65-0.72) | <0.001 | 0.73(0.69-0.78) | <0.001 | 0.75(0.70-0.80) | <0.001 |

^1^Model 1: adjusted for age and sex

^2^Model 2: adjusted for age, sex, SBP, DBP, HbA1c, TC, TG, HDL-C, and LDL-C

^3^Model 3: adjusted for age, sex, SBP, DBP, HbA1c, TC, TG, HDL-C, LDL-C, smoking, and drinking

TFQI, thyroid feedback quantile-based index; PTFQI, parametric thyroid feedback quantile-based index; TSHI, TSH index; TT4RI, thyrotroph thyroxine resistance index; FT3/FT4, free triiodothyronine/ free thyroxine; OR, odd ratio; CI, confidence interval; SBP, systolic blood pressure; DBP, diastolic blood pressure; HbA1c, glycated hemoglobin; TC, total cholesterol; TG, triglycerides; HDL-C, high-density lipoprotein cholesterol; LDL-C, low-density lipoprotein cholesterol.

**Table S3. Association between thyroid hormone sensitivity and the number of carotid plaques**

| **Variables** | **Number of carotid plaques** | | | | | |
| --- | --- | --- | --- | --- | --- | --- |
|  | **1 (*n*=215)** | | | **≥ 2 (*n*=4,628)** | | |
|  | **OR(95 %CI)^1^** | **OR(95 %CI)^2^** | **OR(95 %CI)^3^** | **OR(95 %CI)^1^** | **OR(95 %CI)^2^** | **OR(95 %CI)^3^** |
| TFQI | 23.70(15.45**-**36.37)^**^ | 19.92(12.88**-**30.81)^**^ | 9.52(6.01-15.10)^**^ | 1.84(1.58**-**2.14)^**^ | 1.50(1.28**-**1.76)^**^ | 1.37(1.15**-**1.62)^**^ |
| PTFQI | 43.40(27.49**-**68.52)^**^ | 38.16(23.95**-**60.82)^**^ | 20.12(12.00-33.74)^**^ | 2.12(1.82**-**2.48)^**^ | 1.70(1.45**-**2.00)^**^ | 1.56(1.29-1.88)^**^ |
| TSHI | 3.86(3.17**-**4.71)^**^ | 3.52(2.88**-**4.29)^**^ | 2.82(2.26-3.52)^**^ | 1.35(1.24**-**1.47)^**^ | 1.22(1.12**-**1.34)^**^ | 1.16(1.06-1.28)^**^ |
| TT4RI | 1.02(1.02**-**1.02)^**^ | 1.01(1.01**-**1.02)^**^ | 1.01(1.01**-**1.01)^**^ | 1.01(1.01**-**1.02)^**^ | 1.01(1.00**-**1.01)^**^ | 1.00(1.00-1.01)^*^ |
| FT3/FT4 | 0.04(0.01**-**0.11)^**^ | 0.04(0.01**-**0.11)^**^ | 0.04(0.01**-**0.12)^**^ | 0.72(0.68**-**0.76)^**^ | 0.78(0.74**-**0.83)^**^ | 0.79(0.74**-**0.85)^**^ |

^1^Model 1: adjusted for age, sex;

^2^Model 2: adjusted for age, sex, SBP, DBP, HbA1c, TC, TG, HDL-C, LDL-C;

^3^Model 3: adjusted for sex, SBP, DBP, HbA1c, TC, TG, HDL-C, LDL-C, smoking, drinking.

Compared with no carotid plaques, ^*^*P* < 0.05, ^**^*P* < 0.01.

OR, odds ratio; CI, confidence interval; TFQI, thyroid feedback quantile-based index; PTFQI, parametric thyroid feedback quantile-based index; TSHI, TSH index; TT4RI, thyrotroph thyroxine resistance index; FT3/FT4, free triiodothyronine/free thyroxine; SBP, systolic blood pressure; DBP, diastolic blood pressure; HbA1c, glycated hemoglobin; TC, total cholesterol; TG, triglycerides; HDL-C, high-density lipoprotein cholesterol; LDL-C, low-density lipoprotein cholesterol.

**Table S4. Association between indices of thyroid hormone sensitivity and carotid plaques echogenicity**

| **Variables** | **Carotid plaque echogenicity** | | | | | | | | | | | |
| --- | --- | --- | --- | --- | --- | --- | --- | --- | --- | --- | --- | --- |
|  | **Hypoechoic (*n* = 343)** | | | **Isoechoic(*n* = 307)** | | | **Hyperechoic(*n* = 2,671)** | | | **Mixture(*n* = 1,475)** | | |
|  | **OR(95 %CI)^1^** | **OR(95 %CI)^2^** | **OR(95 %CI)^3^** | **OR(95 %CI)^1^** | **OR(95 %CI)^2^** | **OR(95 %CI)^3^** | **OR(95 %CI)^1^** | **OR(95 %CI)^2^** | **OR(95 %CI)^3^** | **OR(95 %CI)^1^** | **OR(95 %CI)^2^** | **OR(95 %CI)^3^** |
| TFQI | 1.00(0.74-1.34) | 0.83(0.61-1.12) | 0.86(0.62-1.19) | 5.98(4.3-8.24)^**^ | 4.83(3.47-6.72)^**^ | 3.96(2.79-5.64)^**^ | 2.48(2.11-2.93)^**^ | 2.06(1.7-2.44)^**^ | 1.70(1.42-2.05)^**^ | 1.57(1.31-1.89)^**^ | 1.33(1.10-1.61)^**^ | 1.25(1.02-1.54)^*^ |
| PTFQI | 1.063(0.782-1.445) | 0.87(0.63-1.19) | 0.92(0.65-1.30) | 7.80(5.67-10.74)^**^ | 6.33(4.55-8.82)^**^ | 5.54(3.83-8.02)^**^ | 2.90(2.46-3.43)^**^ | 2.37(2.00-2.82)^**^ | 1.95(1.59-2.38)^**^ | 1.80(1.50-2.18)^**^ | 1.51(1.24-1.84)^**^ | 1.43(1.15-1.79)^**^ |
| TSHI | 1.027(0.867-1.216) | 0.94(0.79-1.12) | 0.97(0.81-1.16) | 2.23(1.87-2.67)^**^ | 2.00(1.67-2.40)^**^ | 2.20(1.54-3.14)^**^ | 1.55(1.42-1.71)^**^ | 1.42(1.29-1.56)^**^ | 1.28(1.16-1.42)^**^ | 1.26(1.14-1.40)^**^ | 1.17(1.05-1.30)^**^ | 1.13(1.01-1.27)^**^ |
| TT4RI | 1.00(0.99-1.00) | 0.99(0.99-1.00)^*^ | 0.99(0.99-1.00) | 1.02(1.01-1.02)^**^ | 1.01(1.01-1.02)^**^ | 1.01(1.00-1.01)^**^ | 1.02(1.01-1.02)^**^ | 1.01(1.01-1.01)^**^ | 1.01(1.00-1.01)^**^ | 1.01(1.01-1.01)^**^ | 1.01(1.00-1.01)^**^ | 1.00(1.00-1.01) |
| FT3/FT4 | 0.97(0.87-1.07) | 1.05(0.94-1.17) | 1.03(0.91-1.17) | 0.43(0.39-0.49) | 0.46(0.41-0.53)^**^ | 0.46(0.40-0.53)^**^ | 0.65(0.61-0.68)^**^ | 0.69(0.65-0.74)^**^ | 0.73(0.68-0.79)^**^ | 0.76(0.71-0.81)^**^ | 0.81(0.75-0.86)^**^ | 0.81(0.75-0.88)^**^ |

^1^Model 1: adjusted for age, sex;

^2^Model 2: adjusted for age, sex, SBP, DBP, HbA1c, TC, TG, HDL-C, LDL-C;

^3^Model 3: adjusted for sex, SBP, DBP, HbA1c, TC, TG, HDL-C, LDL-C, smoking, drinking.

Compared with no carotid plaques, ^*^*P* < 0.05, ^**^*P* < 0.01.

OR, odds ratio; CI, confidence interval; TFQI, thyroid feedback quantile-based index; PTFQI, parametric thyroid feedback quantile-based index; TSHI, TSH index; TT4RI, thyrotroph thyroxine resistance index; FT3/FT4, free triiodothyronine/free thyroxine; SBP, systolic blood pressure; DBP, diastolic blood pressure; HbA1c, glycated hemoglobin; TC, total cholesterol; TG, triglycerides; HDL-C, high-density lipoprotein cholesterol; LDL-C, low-density lipoprotein cholesterol.

**Table S5 Linear regression analysis between thyroid hormone sensitivity and CIMT**

| **Variable** | **Model 1 (*n*=4,565)** | | | **Model 2 (*n*=4,565)** | | | **Model 3 (*n*=4,565)** | | |
| --- | --- | --- | --- | --- | --- | --- | --- | --- | --- |
|  | **β** | **95% CI** | ***P*** | **β** | **95% CI** | ***P*** | **β** | **95% CI** | ***P*** |
| TFQI | 0.159 | 0.008-0.011 | <0.001 | 0.149 | 0.008-0.010 | <0.001 | 0.120 | 0.006-0.009 | <0.001 |
| PTFQI | 0.206 | 0.011-0.014 | <0.001 | 0.200 | 0.011-0.014 | <0.001 | 0.181 | 0.009-0.013 | <0.001 |
| TSHI | 0.140 | 0.004-0.006 | <0.001 | 0.132 | 0.004-0.005 | <0.001 | 0.104 | 0.003-0.004 | <0.001 |
| TT4RI | 0.124 | 0.000-0.000 | <0.001 | 0.115 | 0.000-0.000 | <0.001 | 0.085 | 0.000-0.000 | <0.001 |
| FT3/FT4 | -0.235 | -0.005--0.004 | <0.001 | -0.232 | -0.005--0.004 | <0.001 | -0.226 | -0.005--0.004 | <0.001 |

Model 1: adjusted for age and sex

Model 2: adjusted for age, sex, SBP, DBP, HbA1c, TC, TG, HDL-C, and LDL-C

Model 3: adjusted for age, sex, SBP, DBP, HbA1c, TC, TG, HDL-C, LDL-C, smoking, and drinking

TFQI, thyroid feedback quantile-based index; PTFQI, parametric thyroid feedback quantile-based index; TSHI, TSH index; TT4RI, thyrotroph thyroxine resistance index; FT3/FT4, free triiodothyronine/ free thyroxine.

**Table S6 Association between thyroid hormone sensitivity and carotid plaque**

| **Variables** | **Carotid plaque** | | | | | |
| --- | --- | --- | --- | --- | --- | --- |
|  | **OR (95% CI)^1^** | ***P*-value** | **OR (95% CI)^2^** | ***P*-value** | **OR (95% CI)^3^** | ***P*-value** |
| TFQI | 2.09(1.79-2.43) | <0.001 | 1.72(1.47-2.01) | <0.001 | 1.50(1.22-1.73) | <0.001 |
| PTFQI | 2.46(2.11-2.87) | <0.001 | 1.99(1.69-2.34) | <0.001 | 1.71(1.41-2.06) | <0.001 |
| TSHI | 1.44(1.32-1.57) | <0.001 | 1.31(1.19-1.43) | <0.001 | 1.20(1.09-1.32) | <0.001 |
| TT4RI | 1.01(1.01-1.02) | <0.001 | 1.01(1.01-1.01) | <0.001 | 1.00(1.00-1.01) | 0.007 |
| FT3/FT4 | 0.68(0.65-0.72) | <0.001 | 0.73(0.69-0.78) | <0.001 | 0.76(0.71-0.81) | <0.001 |

^1^Model 1: adjusted for age and sex

^2^Model 2: adjusted for age, sex, SBP, DBP, HbA1c, TC, TG, HDL-C, and LDL-C

^3^Model 3: adjusted for age, sex, SBP, DBP, HbA1c, TC, TG, HDL-C, LDL-C, smoking, drinking, and CRP

TFQI, thyroid feedback quantile-based index; PTFQI, parametric thyroid feedback quantile-based index; TSHI, TSH index; TT4RI, thyrotroph thyroxine resistance index; FT3/FT4, free triiodothyronine/ free thyroxine; OR, odd ratio; CI, confidence interval; SBP, systolic blood pressure; DBP, diastolic blood pressure; HbA1c, glycated hemoglobin; TC, total cholesterol; TG, triglycerides; HDL-C, high-density lipoprotein cholesterol; LDL-C, low-density lipoprotein cholesterol; CRP, C-reactionprotein.
